# Monitoring therapy success of urogenital *Chlamydia trachomatis* infections in women: A prospective observational cohort study

**DOI:** 10.1371/journal.pone.0185295

**Published:** 2017-09-21

**Authors:** Bart Versteeg, Sylvia M. Bruisten, Titia Heijman, Wilma Vermeulen, Martijn S. van Rooijen, Alje P. van Dam, Maarten F. Schim van der Loeff, Henry J. C. de Vries, Maarten Scholing

**Affiliations:** 1 Department of Infectious Diseases, Public Health Service Amsterdam, Amsterdam, the Netherlands; 2 Amsterdam Infection and Immunity Institute, Academic Medical Center, University of Amsterdam, Amsterdam, the Netherlands; 3 Department of Medical Microbiology, OLVG General Hospital, Amsterdam, the Netherlands; 4 Department of Dermatology, Academic Medical Center, University of Amsterdam, Amsterdam, the Netherlands; University of California, San Francisco, Universit of California, Berkeley and the Childrens Hospital Oakland Research Institute, UNITED STATES

## Abstract

**Introduction:**

The use of a nucleic acid amplification test (NAAT) as a test of cure after treatment is subject to discussion, as the presence of *C*. *trachomatis* nucleic acids after treatment may be prolonged and intermittent without presence of infectious bacteria. We used cell culture to assess if a positive RNA- or DNA-based NAAT after treatment indicates the presence of viable *C*. *trachomatis*.

**Methods:**

We included women with asymptomatic urogenital *C*. *trachomatis* infection visiting the Amsterdam STI clinic from September 2015 through June 2016. Endocervical swabs were collected prior to treatment with azithromycin, and during three follow-up visits 7, 21 and 49 days after treatment. Collected swabs were subjected to *C*. *trachomatis* culture and a RNA- and DNA-based NAAT. High-resolution multilocus sequence typing (hr-MLST) was used to further differentiate potential re-infections.

**Results:**

We included 90 women with a positive RNA-test prior to receiving treatment of whom 81 (90%) were also DNA-positive, and 69 (76.7%) culture-positive. Prolonged and intermittent positive RNA and DNA results over time were observed. Three women had culture positive results at the second visit, but all were negative at the third visit. Five women had NAAT-positive results at the fourth visit of whom three women were also culture-positive indicating a viable infection. All five women reported unprotected sexual contact since the first visit. From 2, hr-MLST sequence types were obtained. One had a different sequence type indicating a new infection the other was identical to the previously found indicating a potentially persisting infection.

**Conclusion:**

Most RNA- or DNA-positive results after treatment of urogenital *C*. *trachomatis* may be caused by non-viable molecular remnants since they cannot be confirmed by culture. In a minority viable *C*. *trachomatis* was found in culture at the second visit, indicating that patients may remain infectious at least 7 days after treatment.

## Introduction

*Chlamydia trachomatis* infection is a major public health problem, and is the most common bacterial sexually transmitted disease worldwide [1]. *C*. *trachomatis* is capable of infecting various cell types and tissues in the human body with a considerable number of infections found in the urogenital tract. *C*. *trachomatis* primarily infects the columnar epithelial cells of the genital mucosae, with the endocervix being the most commonly infected site in women [2]. Most of these infections remain asymptomatic. If not properly treated, these may result in severe complications including pelvic inflammatory disease, leading to infertility in women and possibly also in men with epididymitis [3–5].

International guidelines recommend nucleic acid amplification tests (NAATs) to diagnose *C*. *trachomatis* due to their superior sensitivity and specificity [2]. The sensitivity of *C*. *trachomatis* cell culture compared to RNA and DNA-based NAATs is low and varies in direct comparisons between 20% to 83% [6–9].

Azithromycin 1000 mg once, is currently the first treatment option for urogenital *C*. *trachomatis* [10]. *C*. *trachomatis* azithromycin treatment is challenged by a growing concern about its efficacy [11–13]. A recent randomized controlled trial did not show inferiority of azithromycin compared to doxycycline in *C*. *trachomatis* [14]. Performing a test of cure (TOC) could demonstrate success or failure of antimicrobial treatment of *C*. *trachomatis*, but the value of using a NAAT (DNA or RNA) based TOC after treatment is subject to discussion, as the presence of *C*. *trachomatis* nucleic acids after treatment may be prolonged and intermittent, and cannot differentiate between dead or viable Chlamydia bacteria [15]. A productive infection after treatment can be proven via the detection of viable bacteria in a positive *C*. *trachomatis* cell culture. The objective of this study was to use cell culture to assess if a positive RNA- or DNA-based NAAT after treatment indicates the presence of viable *C*. *trachomatis*.

## Methods

### Study population

For this study, we included women visiting the STI outpatient clinic of the Public Health Service of Amsterdam, the Netherlands from September 2015 through June 2016. Eligible were women with NAAT-based asymptomatic urogenital *C*. *trachomatis*, 18 years or older and receiving routine azithromycin treatment. Excluded were women with a *C*. *trachomatis* in the three months prior to diagnosis; who received antibiotics three weeks prior to diagnosis; and pregnant women. Women who self-reported anal sex or symptoms were additionally tested for ano-rectal *C*. *trachomatis* and, if positive, were also excluded. Demographic and clinical characteristics of all participating women were obtained from the electronic patient records from the STI outpatient clinic. This study was approved by the Medical Ethics Committee of the Academic Medical Center Amsterdam (NL51851.018.15). All women provided written informed consent.

### Study procedure

Women diagnosed with asymptomatic urogenital *C*. *trachomatis* were asked to return to the STI clinic approximately 7 days after their primary consultation for treatment consisting of a single oral dose of 1000 mg azithromycin. They received information about the study and were invited to participate. Those consenting underwent a speculum examination at the first visit (day 0), prior to receiving treatment, to obtain cervical swabs for *C*. *trachomatis* culture, RNA- and DNA-based NAAT testing. The sampling procedure was repeated at three additional follow-up visits 7, 21 and 49 days after treatment. During each visit, the first collected swab was used for culture. The collection order of swabs for DNA- and RNA-based NAAT testing was reversed on odd and even weeks. Women were requested to abstain from sexual contact or use condoms instead, and refrain from vaginal douching for the duration of the study. Additionally, all were asked to keep a diary on medication use, sexual contact, and vaginal douching during the study period. Women who were NAAT- and/or culture-positive at the third or fourth visit were offered routine re-treatment.

### NAAT testing for *C*. *trachomatis*

Samples for RNA-based NAAT were collected using Aptima endocervical swab specimen kits and tested using the Aptima Combo 2 assay (Hologic Inc, San Diego, California), and relative light units (RLUs) were reported. The instructions of the manufacturer were followed except that all samples with either equivocal or positive results up to 800 RLU for *C*. *trachomatis* were regarded equivocal and were retested using the Aptima CT single assay for confirmation. Repeated equivocal results were considered positive for the analysis. Samples with repeated invalid results were excluded.

Samples for DNA-based NAAT were collected using Cobas polymerase chain reaction (PCR) female swab kits and were tested using the Cobas 4800 assay for *C*. *trachomatis* and *N*. *gonorrhoeae* (Roche, Basel, Switzerland), and the cycle threshold (Ct) was reported for positive samples. Women that tested NAAT-negative for *C*. *trachomatis* at the first visit were excluded from further analysis.

### *C*. *trachomatis* culture

For *C*. *trachomatis* culture, endocervical swabs were collected from women and stored in 3 ml universal transport medium (UTM-RT, Copan Italia S.p.A., Brescia, Italy). Collected samples were immediately stored at -80°C until further use. *C*. *trachomatis* culture was performed on all samples at the first visit, and on all samples from follow-up visits with a positive RNA- and/or DNA-based NAAT result. Cell culture procedures were adapted from Isenberg [16]. In short, HeLa 229 cells were cultured in Iscoves Modified Dulbecco's Medium (IMDM; Gibco life technologies, Germany) supplemented with 10% fetal bovine serum (Lonza Bio Science, Verviers, Belgium) at 37°C. Then, 300μl of swab medium was inoculated on a HeLa 229 monolayer present on a round coverslip in a flat bottomed tube with IMDM medium supplemented with 10% fetal bovine serum (Lonza Bio Science, Vierviers, Belgium), 4.5 g/L glucose, 2.5 μg/ml amphotericin B and 10 μg/ml gentamicin (Gibco life technologies, Germany). Infection was completed by centrifugation-assisted inoculation at 3400 rpm for 1h at 34°C, and subsequently cells were incubated at 34°C. Each sample was cultured in duplicate. One coverslip was analysed after 72h by immunofluorescence (IF) staining to identify viable chlamydial bacteria. Negative samples were subpassaged to a new coverslip containing HeLa 229 cells and incubated for another 72h and examined by IF.

### Immunofluorescence staining

Direct IF staining was performed on methanol fixed inoculated monolayers using a fluorescein isothiocyanate (FITC) conjugated, mouse anti-chlamydia monoclonal antibody targeting the chlamydial lipopolysaccharide antigen (OXOID Limited, Hampshire, United Kingdom), according to the manufacturer's instructions. The specimens were examined at a magnification of 400x with a fluorescence microscope (Leica Microsystems GmbH, Wetzlar, Germany). Typically, specimens displayed multiple red-fluorescent stained HeLa 229 cells. Positive appearing samples were further examined at a magnification of 1000x. Green-fluorescent stained Chlamydial intracytoplasmic inclusions were counted for all specimens. Slides were scored as positive if 10 distinct fluorescein-stained intracytoplasmic inclusions were observed.

### High-resolution MLST

*C*. *trachomatis* strains in NAAT- or culture-positive results after treatment were further analysed using high-resolution multilocus sequence typing (hr-MLST) [17–19]. Sequence types (STs) of the strains determined at the first and fourth visit were compared. In case non-identical STs were found we concluded that a re-infection had occurred. In case identical STs were found we deduced that either a re-infection or persistent infection had occurred. For hr-MLST, DNA extracts were amplified by a nested PCR and sequenced for the regions ompA, CT046 (hctB), CT058, CT144, CT172, and CT682 (pbpB). The primer-to-primer sequences were checked against the *Chlamydia trachomatis* hr-MLST database (http://mlstdb.bmc.uu.se). Only samples in which all six loci were successfully amplified, sequenced, and identified obtained a full hr-MLST sequence type.

### Statistical analysis

The primary endpoint, clearance of *C*. *trachomatis* RNA, DNA or viable bacteria (culture), was defined as a negative result for each individual assay at any visit after treatment. Chi-square and Fisher exact tests were used to compare categorical variables between groups; the Kruskal-Wallis test was used to compare continuous variables between groups, and *P* <0.05 was considered statistically significant. Time to clearance was analysed with Kaplan-Meier curves. Clearance was assumed to have occurred at the day of the first negative test. All statistical analyses were performed using SPSS version 21.0 (SPSS Inc., Chicago, IL, USA) and STATA Intercooled V.11.0 (STATA, College Station, Texas, USA)

## Results

### Study population

**Fig 1:**
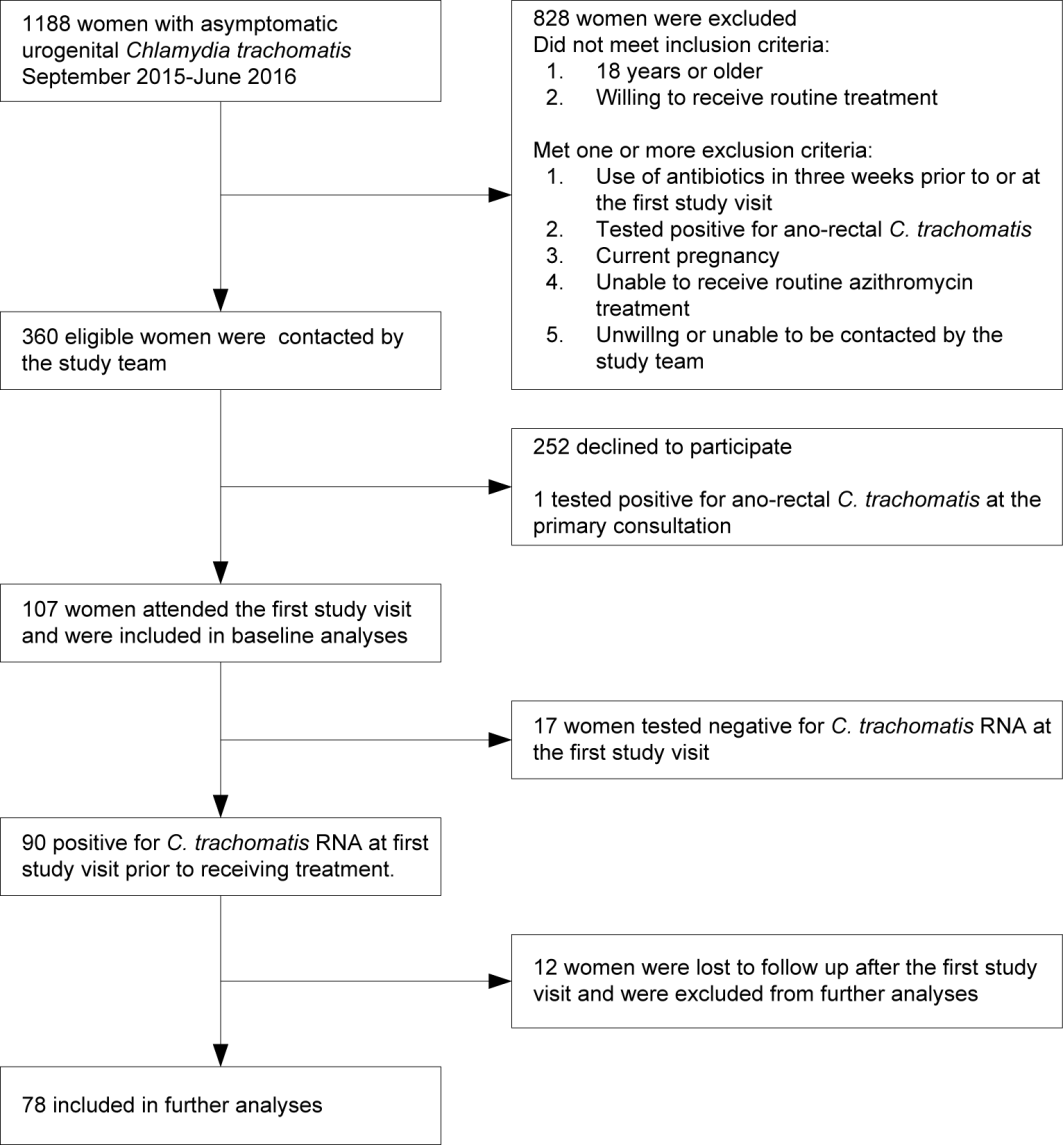
Flowchart of the study population. Flowchart indicating the number of women included and excluded from the study population. Amsterdam 2015–2016.

From September 2015 through June 2016, 1188 female were diagnosed with asymptomatic urogenital *C*. *trachomatis*, 360 eligible women were invited to participate, and 108 women consented to participate. One woman was diagnosed with a concurrent ano-rectal *C*. *trachomatis* and therefore excluded, resulting in a total of 107 women who completed the first visit and were included in baseline analyses (Fig 1). From these, 17 women self-cleared their infection prior to the first visit, and 12 women were lost to follow-up after the first visit. Since we could not determine actual clearance of the infection after treatment, or determine potential treatment-failure, re-infection or persisting infections in these women, they were all excluded from further analyses. Follow-up visits were scheduled 7, 21 and 49 days after treatment, and were attended by all 78 remaining women (72.9%). These visits were a median 7 (IQR 7–7), 22 days (IQR 21–25) and 49 days (IQR 49–52) after the first visit. The longest period between the first and fourth study visit was 69 days.

### Baseline characteristics of included women stratified by NAAT-testing

The median age of the 107 included women was 22 years (interquartile range [IQR]: 21–24 years) and the median time between their primary consultation and the first visit was 9 days (IQR: 7–12 days, Table 1). Of those, 90 (84.1%) were still RNA-positive prior to treatment at the first visit, and 81(75.7%) were also DNA-positive; all DNA-positive women were also RNA positive. The remaining 17 (15.9%) were RNA-negative at the first visit, suggesting self-clearance of the infection. Overall, no significant differences were observed between women who self-cleared their initial infection compared to those who remained NAAT-positive at the first visit (Table 1).

**Table 1:** Characteristics of 107 women according to their NAAT result at inclusion. Amsterdam STI clinic, 2015–2016.

Characteristic	Total		*C*. *trachomatis* RNA-positive		*C*. *trachomatis* RNA-negative		*P*-value
	n	(%)	n	(%)	n	(%)	
Included women	107	(100.0%)	90	(84.1%)	17	(15.9%)	-
Age in years (continuous)							0.416
Median (IQR)	22	(21–24)	22	(21–24)	21	(21–23)	
Ethnicity							0.928
Dutch	64	(59.8%)	54	(60.0%)	10	(58.8%)	
Non-Dutch	43	(40.2%)	36	(40.0%)	7	(41.2%)	
Notified for STI by a sexual partner							0.342
No	100	(93.5%)	85	(94.4%)	15	(88.2%)	
Yes	7	(6.5%)	5	(5.6%)	2	(11.8%)	
Time from pre-visit to first visit in days							0.274
Median (IQR)	9	(7–12)	9	(7–12)	11	(8–14)	
RLU-value pre-treatment ^a^							-
Median (IQR)	1204	(1130–1286)	1204	(1130–1286)	-	-	
Ct-value pre-treatment ^b^							-
Median (IQR)	30.4	(27.4–32.6)	30.4	(27.4–32.6)	-	-	
*C*. *trachomatis* culture							<0.001
Negative	36	(33.6%)	21	(23.3%)	16	(94.1%)	
Positive	69	(64.5%)	69	(76.7%)	0	(0.0%)	
Failed ^c^	2	(1.9%)	0	(0.0%)	1	(5.9%)	
History of *C*. *trachomatis* ^d^							0.741
No or Unknown	85	(79.4%)	72	(80.0%)	13	(76.5%)	
Yes	22	(20.6%)	18	(20.0%)	4	(23.5%)	
Last known sexual partner							0.288
Casual partner	57	(53.3%)	47	(52.2%)	10	(58.8%)	
Steady partner	28	(26.2%)	26	(28.9%)	2	(11.8%)	
Unknown	22	(20.6%)	17	(18.9%)	5	(29.4%)	

Data are presented as No. (%) unless otherwise indicated.

Abbreviations: IQR, interquartile range; STI, sexually transmitted infections; Ct, cycle threshold; RLU, relative light unit; NAAT, nucleic acid amplification test.

^a^ Including samples positive for C. trachomatis RNA (n = 90).

^b^ Including samples positive for C. trachomatis DNA (n = 81).

c Failed due to contamination of other micro-organisms present in the sample.

^d^ History of C. trachomatis refers to either a self-reported or previously documented C. trachomatis infection >3 months prior to inclusion.

### Baseline characteristics of *C*. *trachomatis* NAAT-positive women stratified by culture

A positive culture result was found for 69 of the 90 (76.7%), RNA-positive, and 63 of the 81 (77.8%) DNA-positive women (Table 2). Comparison of characteristics between women with a positive or negative *C*. *trachomatis* culture revealed that women with a negative *C*. *trachomatis* culture had significantly lower median RLU-values pre-treatment (median RLU-value 1100) compared to women with a positive *C*. *trachomatis* culture (median RLU-value 1219, *P* = 0.002). In addition, women with a negative *C*. *trachomatis* culture had higher median Ct-values pre-treatment (median Ct-value 34.6) compared to women with a positive *C*. *trachomatis* culture result (median Ct-value 29.3, *P <*0.001). Both findings indicate that samples from women with a negative culture had a lower chlamydial load.

**Table 2:** Characteristics of 90 NAAT-positive women according to their *C*. *trachomatis* culture result. Amsterdam STI clinic, 2015–2016.

Characteristic	Total		*C*. *trachomatis* culture positive		*C*. *trachomatis* culture negative		*P*-value
	n	(%)	n	(%)	n	(%)	
Included women	90	(100.0%)	69	(76.7%)	21	(23.3%)	-
Age in years (continuous)							0.143
Median (IQR)	22	(21–24)	22	(20–24)	23	(21–27)	
Ethnicity							0.161
Dutch	55	(61.1%)	45	(65.2%)	10	(47.6%)	
Non-Dutch	35	(38.9%)	24	(34.8%)	11	(52.4%)	
Notified for STI by a sexual partner							0.063
No	85	(94.4%)	68	(98.6%)	17	(81.0%)	
Yes	5	(5.6%)	1	(1.4%)	4	(19.0%)	
Time from pre-visit to first visit in days							0.155
Median (IQR)	9	(7–12)	9	(7–13)	8	(7–12)	
RLU-value pre-treatment ^a^							0.002
Median (IQR)	1200	(1129–1285)	1219	(1148–1301)	1100	(770–1224)	
*C*. *trachomatis* DNA							0.046
Positive	80	(88.9%)	63	(91.3%)	17	(81.0%)	
Negative	9	(10.0%)	6	(8.7%)	4	(19.0%)	
Ct-value pre-treatment ^b^							<0.001
Median (IQR)	30.4	(27.4–32.7)	29.3	(26.5–31.1)	34.6	(31.7–36.7)	
History of *C*. *trachomatis* ^c^							0.063
Yes	73	(81.1%)	53	(76.8%)	20	(95.2%)	
No or unknown	17	(18.9%)	16	(23.2%)	1	(4.8%)	
Last known sexual partner							0.559
Casual partner	47	(52.2%)	37	(53.6%)	10	(47.6%)	
Steady partner	26	(28.9%)	18	(26.1%)	8	(38.1%)	
Unknown	17	(18.9%)	14	(20.3%)	3	(14.3%)	

Data are presented as No. (%) unless otherwise indicated.

Abbreviations: IQR, interquartile range; STI, sexually transmitted infections; Ct, cycle threshold; RLU, relative light unit; NAAT, nucleic acid amplification test.

^a^ Including samples positive for C. trachomatis RNA (n = 90).

^b^ Including samples positive for C. trachomatis DNA (n = 81).

^c^ History of C. trachomatis refers to either a self-reported or previously documented C. trachomatis infection >3 months prior to inclusion.

### Clearance of *C*. *trachomatis* RNA and DNA

**Fig 2:**
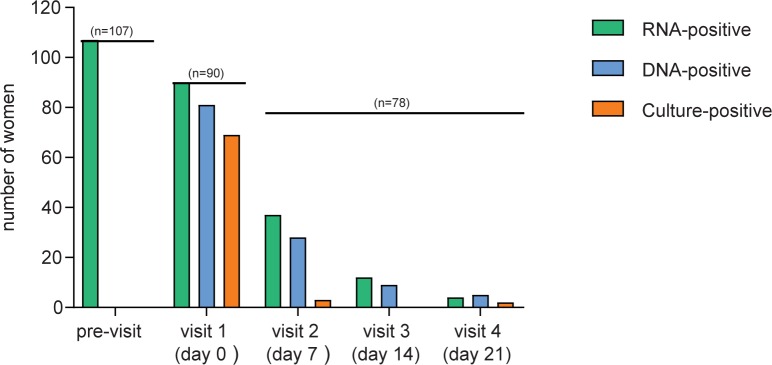
The number of women that tested positive for *Chlamydia trachomatis* per test visit. Graph showing the total number of women that tested positive for *C*. *trachomatis* RNA, DNA or culture. Lines indicate the total number of women included at each visit.

**Fig 3:**
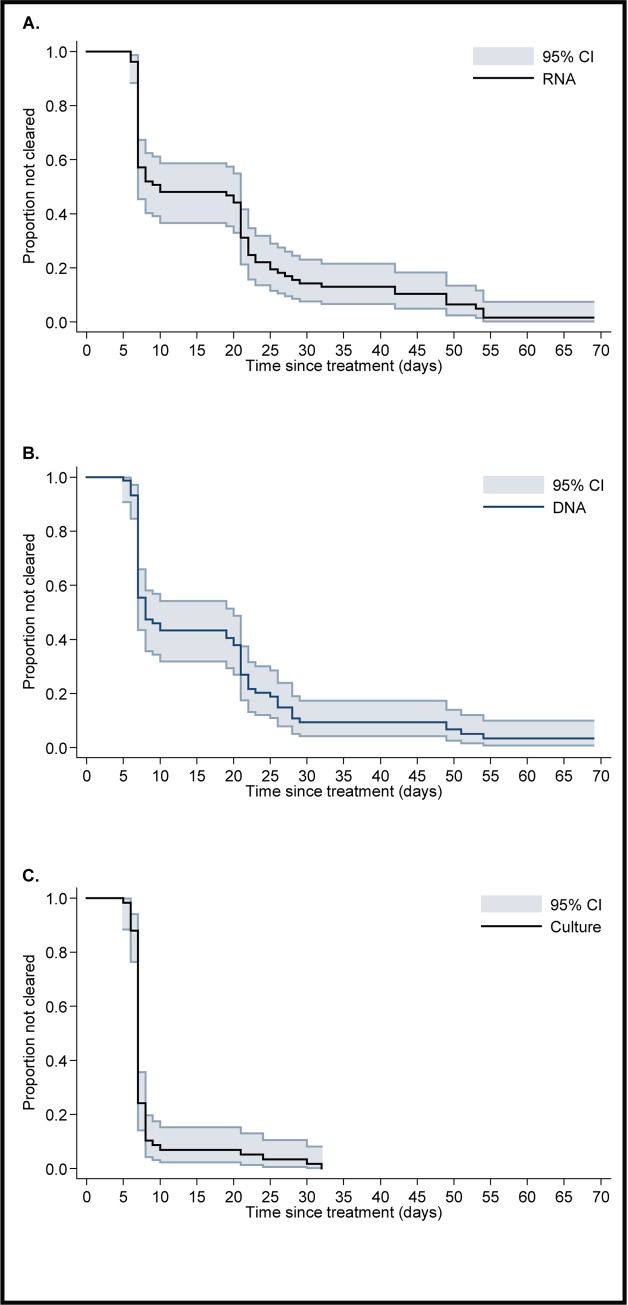
Time to clearance of *Chlamydia trachomatis* RNA, DNA and culture. (A) Time to clearance of *Chlamydia trachomatis* RNA (n = 78), (B) time to clearance of *Chlamydia trachomatis* DNA (n = 70) and (C) time to clearance of *Chlamydia trachomatis* culture (n = 58) with 95% confidence interval (CI).

The number of participating women with positive RNA or DNA results at each visit is shown in Fig 2 and 78 had complete follow-up. Of these 78 women 52.6% (n = 41) cleared RNA at the second visit, 84.6% (n = 66) at their third visit, and 97.4% (n = 76) at the fourth visit (Table 3 and Fig 3A). Of the women who had initially cleared RNA, two became RNA-positive again during follow-up visits.

**Table 3:** Behaviour of women after inclusion and clearance of *Chlamydia trachomatis* infection based on testing by RNA, DNA and culture.

Characteristic	RNA-positive		DNA-positive		Culture-positive	
	n = 78 women		n = 70 women		n = 58 women	
	n	(%)	n	(%)	n	(%)
Behaviour after inclusion						
Vaginal douching	9	(11.5%)	9	(12.9%)	8	(13.8%)
Sexual contact	59	(75.6%)	54	(77.1%)	43	(74.1%)
Unprotected sexual contact	41	(52.6%)	37	(52.9%)	32	(55.2%)
Visit 2						
Time since visit 1 in days (median, IQR)	7	(7–7)	7	(7–7)	7	(7–7)
Remained *C*. *trachomatis* positive since treatment	37	(47.4%)	28	(40.0%)	3	(5.2%)
Clearance since treatment	41	(52.6%)	42	(60.0%)	55	(94.8%)
Visit 3						
Time since visit 1 in days (median, IQR)	22	(21–25)	22	(21–25)	22	(21–25)
Remained *C*. *trachomatis* positive since treatment	10	(12.8%)	7	(10.0%)	0	(0.0%)
Cleared since treatment	66	(84.6%)	61	(87.1%)	58	(100.0%)
Women tested positive again after initial clearance	2	(2.6%)	2	(2.9%)	0	(0.0%)
Visit 4						
Time since visit 1 in days (median, IQR)	49	(49–52)	49	(49–52)	50	(49–53)
Remained *C*. *trachomatis* positive since treatment	2	(2.6%)	3	(4.3%)	0	(0.0%)
Cleared since treatment	74	(94.9%)	65	(92.9%)	56	(96.6%)
Women tested positive again after initial clearance	2	(2.6%)	2	(2.9%)	2	(3.4%)

Data are presented as No. (%) unless otherwise indicated.

Abbreviations: IQR, Interquartile range

**Fig 4:**
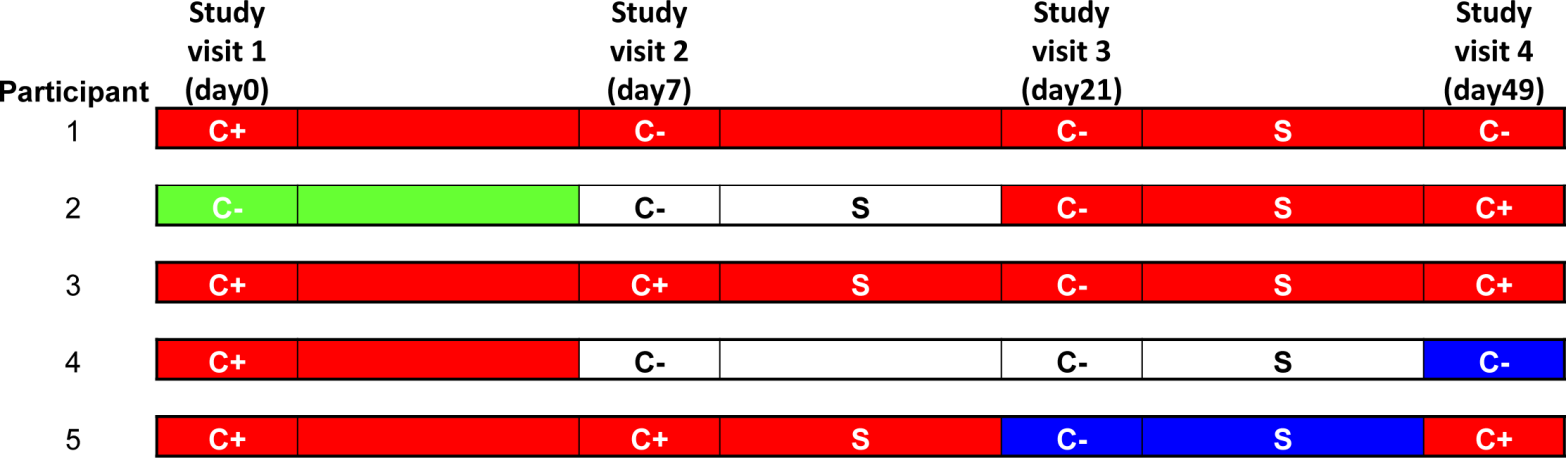
Overview of women tested DNA, RNA or Culture positive at the fourth visit. *Chlamydia trachomatis* RNA and DNA-based NAAT test results, sexual contact and culture result per visit for 5 women with positive results at the fourth visit. Red: DNA- and RNA-positive; green: RNA-positive and DNA-negative; blue, DNA-positive and RNA-negative; White: RNA- and DNA-negative; C+: culture positive; C- culture negative; S: unprotected sexual contact reported after the previous visit.

Of 70 (92.9%) women with a DNA-positive NAAT, 68 cleared DNA after treatment at some visit before their fourth visit (Table 3 and Fig 3B); 60% (n = 42) cleared DNA at the second visit and 87.1% (n = 61) at the third visit. Three women (2.9%) remained DNA-positive after treatment of whom 2 also remained RNA-positive (Fig 4, patients 1 and 3). Of the 68 women who cleared, two tested DNA-positive again during follow-up visits.

In total, five women were NAAT-positive at their fourth visit; 4 both DNA- and RNA-positive and one only DNA-positive (Fig 4). All 5 reported unprotected sexual contact after treatment.

### *C*. *trachomatis* cell culture

The number of all participating women with a positive culture result is shown for each visit in Fig 2. *C*. *trachomatis* culture was positive for 58/78 (74.4%) women with complete follow up and that were NAAT-positive at their first visit. Of those 3 remained culture-positive at their second visit, none reported unprotected sexual contact, 2 had returned for their second visit 7 days after azithromycin intake. The moment of azithromycin intake of the remaining woman could not be established. All three women cleared their infection by the third visit without additional treatment (Table 3 and Fig 3C).

Three women were culture-positive at their fourth visit of whom two initially cleared their positive culture (Fig 4, patient 3 and 5). The other woman (Fig 4, patient 2) was culture negative at the first visit but became positive at the fourth visit. All three women reported unprotected sexual contact and had detectable *C*. *trachomatis* RNA and DNA (Fig 4).

### High-resolution MLST

Hr-MLST typing was successful on 2/5 sets of samples from the first and fourth visit of the 5 women with persistent NAAT- and/or culture-positive results. For patient 3 (Fig 4), the sample from the first visit showed a sequence type 55 (ST55) whereas in her sample from the fourth visit ST13b was found. This indicates a new infection. For patient 4, both the samples from the first and fourth visit showed ST441, indicating either a persistent infection that was too low to be detected at interim visits or re-infection.

## Discussion

In the present study we compared RNA- and DNA-based NAATs with conventional cell culture to assess whether a positive NAAT-based test of cure indicates the presence of viable *C*. *trachomatis* bacteria in urogenital *C*. *trachomatis* after routine azithromycin treatment. Overall, we observed prolonged and intermittent positive results over time for RNA- and DNA-based NAATs during follow-up visits for some women. In 76.7% (69/90) RNA-positive and 77.8% (63/81) DNA-positive women, a viable *C*. *trachomatis* was found at the first visit. Three women had a positive culture at the second visit, but all became negative at the third visit.

Seventeen women (15.9%) self-cleared their infection, prior to receiving treatment, after a median period of 9 days. This proportion is comparable to other studies that reported approximately 20% of spontaneous self-clearance within 2 to 3 weeks [20–23]. It is hypothesized that spontaneous clearance is associated with developing protective immunity against *C*. *trachomatis* [21]. All women with self-cleared infections had equivocal results in the Aptima combo test at primary consultation, but were confirmed positive upon retesting using the Aptima CT single assay.

We found intermittent positive results for DNA- and RNA-based NAATs up to 49 days in 5% of participants which is much lower compared to a previous study where intermittent positive results for DNA- and RNA-based NAATs were found in up to 42% of participants up to 51 days after treatment [15]. For DNA, three women (2.9%) remained DNA-positive, of whom two also remained RNA-positive throughout the study period up to the fourth visit. In addition, two women became RNA-positive again and one also DNA-positive after negative RNA and/or DNA-based NAATs at a previous visit. One woman became only DNA-positive again after negative RNA and/or DNA-based NAATs at a previous visit. Previous studies reported 12% RNA persistence after 4 weeks in men [24], and 24% RNA positivity in patients returning to an STI clinic after 6 months [25]. For DNA clearance other, mostly older studies, reported contradictory results; some found clearance within 3 weeks while others reported DNA persistence in 5–25% of the patients after 3–4 weeks [15, 26–28]. In a previous study, we found that most (*N*. *gonorrhoeae* co-infected) patients cleared *C*. *trachomatis* DNA and RNA within 14 days [29]. In contrast to the current study, none of these previous studies used *C*. *trachomatis* culture to provide additional information on RNA- or DNA-based NAAT-positive results. *C*. *trachomatis* culture may distinguish between viable *C*. *trachomatis* and RNA or DNA remnants.

Despite lower sensitivity compared to NAATs, a positive culture is the most specific method available to detect a viable infection and may therefore help to interpret (remnant) positive NAAT results. The reported sensitivity of *C*. *trachomatis* culture compared to NAAT testing varies in direct comparisons between 20% to 83% [6–9]. In our study, we observed a sensitivity of 76.7% (69/90) for culture in comparison to the Aptima RNA-based NAAT, and 77.8% (63/81) sensitivity for culture in comparison to the Cobas DNA-based NAAT. In addition, we compared characteristics of women with a culture-positive and culture-negative infection and observed that culture-negative samples had a lower Chlamydial load as indicated by the significantly lower RLU and higher Ct-values.

Using *C*. *trachomatis* culture, we found that three women still had a positive culture sample at their second visit None of these women reported unprotected prior sexual contact, suggesting that a minority of cases may remain infectious at least 7 days after treatment with a single azithromycin dosage. For none of the participants included in the study the azithromycin intake was directly observed which is in accordance to routine practice at the Amsterdam STI clinic. Two of three women returned for their second visit at 7 days after self-reported azithromycin intake. The moment of azithromycin intake of the remaining woman could not be established. Delayed intake of azithromycin could have resulted in very high initial bacterial loads that cleared more slowly. It could also be that these women did have unprotected sexual contact but failed to report so, or that Chlamydia bacteria persisted in mucosal cells that were cleared more slowly than expected as *C*. *trachomatis* is known to form enlarged aberrant non-dividing reticulate bodies *in vitro* in the presence of antibiotics [30]. This process is reversible after removal of the antibiotics allowing the *C*. *trachomatis* to differentiate in infectious elementary bodies, again causing persisting infections. However, this is unlikely since all culture results were negative at the third visit.

Five women tested DNA and/or RNA-positive again at their last visit of whom three tested also culture-positive, indicating a viable infection, after initially having cleared. All three women reported unprotected prior sexual intercourse suggesting that these were possibly re-infections. For two of five women hr-MLST sequence types (ST) were obtained from the first and fourth visit samples; one had different STs indicating most likely a re-infection. The other had identical STs, likely caused by re-infection, from the same (possibly untreated) partner. With exception of the infection caused by different STs, it is of course possible that these infections are persisting viable infections that were not detected since culture lacks sensitivity to detect all viable chlamydial infections. Therefore, we might have underestimated the number of persisting infections after treatment during the study period, which could suggest that more participants remained infectious after treatment. However, since most women (76/78; 97.4%) cleared both chlamydial DNA and RNA sometime after treatment and prior to the fourth study visit, we think it unlikely that these constitute persisting infections. Another explanation could be ano-rectal to urogenital autoinoculation [31, 32]. Since we did not test all participants for rectal *C*. *trachomatis*, we cannot rule out autoinoculation from a sub-optimally (azithromycin once) treated anorectal infection.

Potential limitations of this study should be noted. We studied high-risk patients who may not be representative for the general *C*. *trachomatis*-infected population. We had no data on the sexual partners of the included women and could therefore not determine whether they received appropriate care to prevent re-infection after treatment. In addition, we could only type 2 of 5 paired samples with possible re-infections using hr-MLST, due to low chlamydial load. Because of a limited budget we collected swabs on four visits with weeks apart and not more frequently, so we were not able to determine a more exact moment of clearance in these women.

We observed prolonged and intermittent positive results over time for RNA- and DNA-based NAATs, yet the large majority did not correspond to viable infections. Viable *C*. *trachomatis* were found at least 7 days after treatment, indicating that potential forward transmission within this period is feasible after receiving azithromycin once. For three women, *C*. *trachomatis* cultures were positive at the fourth visit approximately 49 days after treatment. These were considered likely re-infections from (untreated) partners. Overall, we observed that a positive NAAT after treatment does not necessarily indicate treatment failure or re-infection. We have not found a clear indication for persisting infections or treatment failure.
